# Genetic suppression reveals DNA repair-independent antagonism between BRCA1 and COBRA1 in mammary gland development

**DOI:** 10.1038/ncomms10913

**Published:** 2016-03-04

**Authors:** Sreejith J. Nair, Xiaowen Zhang, Huai-Chin Chiang, Md Jamiul Jahid, Yao Wang, Paula Garza, Craig April, Neeraj Salathia, Tapahsama Banerjee, Fahad S. Alenazi, Jianhua Ruan, Jian-Bing Fan, Jeffrey D. Parvin, Victor X. Jin, Yanfen Hu, Rong Li

**Affiliations:** 1 Department of Molecular Medicine, The University of Texas Health Science Center at San Antonio, San Antonio, Texas 78229, USA; 2 Department of Computer Science, The University of Texas at San Antonio, San Antonio, Texas 78249, USA; 3 Research and Development, Illumina, Inc., San Diego, California 92122, USA; 4 Department of Biomedical Informatics, The Ohio State University, Columbus, Ohio 43210, USA

## Abstract

The breast cancer susceptibility gene *BRCA1* is well known for its function in double-strand break (DSB) DNA repair. While BRCA1 is also implicated in transcriptional regulation, the physiological significance remains unclear. COBRA1 (also known as NELF-B) is a BRCA1-binding protein that regulates RNA polymerase II (RNAPII) pausing and transcription elongation. Here we interrogate functional interaction between BRCA1 and COBRA1 during mouse mammary gland development. Tissue-specific deletion of *Cobra1* reduces mammary epithelial compartments and blocks ductal morphogenesis, alveologenesis and lactogenesis, demonstrating a pivotal role of COBRA1 in adult tissue development. Remarkably, these developmental deficiencies due to *Cobra1* knockout are largely rescued by additional loss of full-length *Brca1*. Furthermore, *Brca1*/*Cobra1* double knockout restores developmental transcription at puberty, alters luminal epithelial homoeostasis, yet remains deficient in homologous recombination-based DSB repair. Thus our genetic suppression analysis uncovers a previously unappreciated, DNA repair-independent function of BRCA1 in antagonizing COBRA1-dependent transcription programme during mammary gland development.

## Abstract (web summary)

COBRA1 is a BRCA1-binding protein and, as part of the negative elongation factor, regulates RNA polymerase II pausing and transcription elongation. Here, the authors show that tissue-specific deletion of mouse *Cobra1* inhibits postnatal mammary gland development and that the mammary defects can be rescued by additional deletion of *Brca1* in a DNA repair-independent manner.

## Graphical abstract

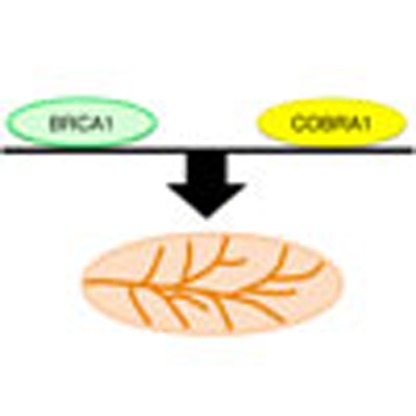

Germline mutations in *BRCA1* predispose women to breast and ovarian cancers^1^. BRCA1 is best known for its role in promoting the homologous recombination (HR)-based pathway of DNA double-strand break (DSB) repair^2^. In addition to DSB repair, BRCA1 has also been implicated in other cellular processes, including transcription^3^,^4^. BRCA1 binds to RNA polymerase II (RNAPII)^5^ and various site-specific transcription factors, including oestrogen receptor α (ERα) and GATA3 (refs 6, 7), which are involved in mammary gland development and breast cancer. Our previous work indicates that BRCA1 is capable of regulating transcription through high-order chromatin reorganization and unfolding^8^,^9^,^10^,^11^,^12^. Consistent with a role for BRCA1 in transcriptional regulation, genome-wide analysis indicates that chromatin binding of BRCA1 is enriched at transcription start sites (TSS) across the human genome^13^,^14^,^15^. Notwithstanding these *in vitro* findings, there is a gap of knowledge concerning the physiological relevance of these transcription-related activities of BRCA1 *in vivo*.

We previously identified a BRCA1-binding protein, cofactor of BRCA1 or COBRA1 (ref. 11), which is identical to the B subunit of the negative elongation factor (NELF) complex^16^. NELF is a metazoan-specific regulator of transcription elongation that pauses RNAPII at the TSS-proximal region^17^,^18^. Although NELF was first identified as a transcription elongation repressor *in vitro*^16^, subsequent *in vivo* studies indicate that NELF-mediated RNAPII pausing can lead to both decreased and increased transcription^18^,^19^,^20^,^21^. In ERα^+^ breast cancer cells, COBRA1 interacts with ERα (ref. 22) and regulates RNAPII movement at ERα target genes^23^,^24^. While NELF-mediated RNAPII pausing has been proposed to ensure synchronous transcriptional activation of developmentally regulated genes^25^, the exact physiological roles of mammalian NELF have just begun to be deciphered. Published work from us and others indicates that mouse COBRA1 is critical for early embryogenesis^26^,^27^ and energy homoeostasis in adult myocardium^28^. However, the role of COBRA1 in adult tissue development remains unexplored. Furthermore, it is not clear whether the previously characterized physical interaction between COBRA1 and BRCA1 has any physiological significance. Using mammary epithelium-specific knockout (KO) mouse models for *Brca1* and *Cobra1*, we provide compelling genetic evidence for a previously unrecognized functional link between BRCA1 and a transcription elongation factor in dictating the developmental outcome in mammary epithelium.

## Results

### *Brca1* and *Cobra1* complementation in ductal development

**Figure 1:**
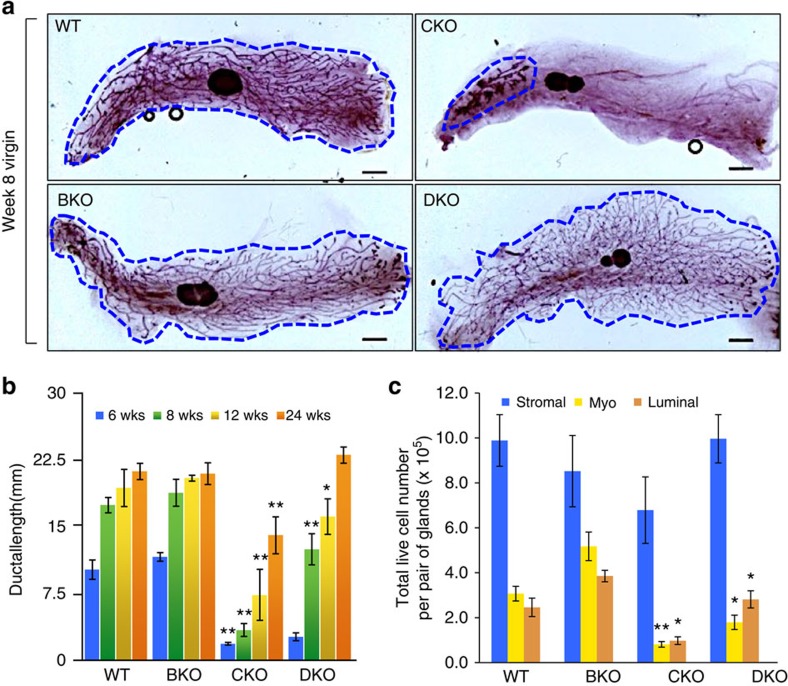
DKO rescues ductal developmental defect in CKO. (**a**) Whole mounts of mammary glands from 8-wk virgin mice. The boundary of the ductal area is highlighted. Scale bars, 1 mm. Images are representatives of at least 6 animals. (**b**) Measurement of the average ductal length at four developmental time points. The numbers of animals used for each of the four time points (6, 8, 12 and 24 wks) are: WT (4, 7, 7 and 12 mice), BKO (3, 3, 4 and 4 mice), CKO (3, 6, 5 and 8 mice) and DKO (4, 7, 4 and 4 mice). Statistical analyses here and in **c** were conducted using Student's *t*-test for comparison between CKO and WT, and between DKO and CKO. (**c**) Flow cytometry analysis of total live cells in various subpopulations per mammary gland from 16-wk virgin mice. Stromal cells: CD49f^-^EpCAM^−^, luminal epithelial cells: CD49f^med^EpCAM^high^, myoepithelial cells: CD49f^high^EpCAM^med^. The numbers of animals used are: WT (4), BKO (3), CKO (3) and DKO (4). *P<0.05, **P<0.01 by Student's *t*-test. Error bars represent s.e.m. wks, weeks.

To investigate the role of COBRA1 in mammary gland development, we generated mammary epithelium-specific KO mice by breeding the *MMTV-Cre* strain^29^ with *Cobra1*^*f/f*^ animals^26^, which resulted in deletion of the first four *Cobra1* exons. Mammary epithelium of the resulting female *MMTV-Cre*,*Cobra1*^*f/f*^ (CKO) animals was effectively depleted of COBRA1 (Supplementary Fig. 1a,b). Compared with age-matched wild-type littermates (WT, *Cobra1*^*f/f*^) and hemizygous mice (*MMTV-Cre*,*Cobra1*^*f/+*^), virgin CKO with homozygous deletion of *Cobra1* displayed severely retarded mammary ductal growth (Fig. 1a,b; Supplementary Fig. 2a). The normal developmental phenotype of hemizygous *Cobra1* knockout mice (*MMTV-Cre,Cobra1*^*f/+*^, Supplementary Fig. 2a) strongly indicates that the CKO-associated developmental defects are due to homozygous *Cobra1* ablation, not Cre recombinase expression *per se*. We therefore used littermates carrying floxed WT gene alleles as the control for most of our subsequent experiments.

The developmental defect of CKO was most profound during and shortly after puberty (6 and 8 weeks), and remained significant in older virgin mice (12 and 24 weeks, Fig. 1b). In further support, flow cytometry using established cell-surface markers for mammary epithelial cells^30^ showed that total live luminal (CD49f^med^EpCAM^high^) and myoepithelial (CD49f^high^EpCAM^med^) cell populations of CKO mammary glands were equally reduced compared with their WT littermates (Fig. 1c; Supplementary Fig. 3), suggesting an overall developmental arrest of multiple mammary epithelial lineages. These data clearly indicate that mouse COBRA1 is important for adult tissue development.

Given the physical interaction between BRCA1 and COBRA1 (ref. 11), we compared the phenotypes of CKO with *MMTV-Cre*-mediated *Brca1* KO that was conditionally deleted of *Brca1* exon 11 (*MMTV-Cre,Brca1*^*f/f*^; BKO), and *Brca1/Cobra1* double KO mice (*MMTV-Cre,Brca1*^*f/f*^*,Cobra1*^*f/f*^; DKO). Consistent with published findings^31^,^32^, BKO animals exhibited grossly normal ductal growth at puberty (Fig. 1a,b). Ductal development of DKO mice was stunted at 6 weeks (Fig. 1b; Supplementary Fig. 4), but remarkably, it approached that of WT and BKO at later stages (Fig. 1a,b). Furthermore, abundance of luminal and myoepithelial cells in DKO mammary glands was significantly higher than that in age-matched CKO (Fig. 1c). While myoepithelial cell number in DKO was still lower than that in WT (*P*=0.026 by Student's *t*-test), there was no appreciable difference in luminal cell abundance between DKO and WT. We confirmed that COBRA1 and BRCA1 expression in DKO mice were depleted to a similar extent versus the corresponding single-gene KO animals (Supplementary Fig. 1b,c). Therefore the marked phenotypic difference between CKO and DKO reflects a *bona fide* genetic complementation between *Brca1* and *Cobra1*. We interpret our finding to mean that BRCA1 imposes a developmental blockade on CKO mammary glands.

### Rescue of alveologenesis and lactogenesis in DKO

**Figure 2:**
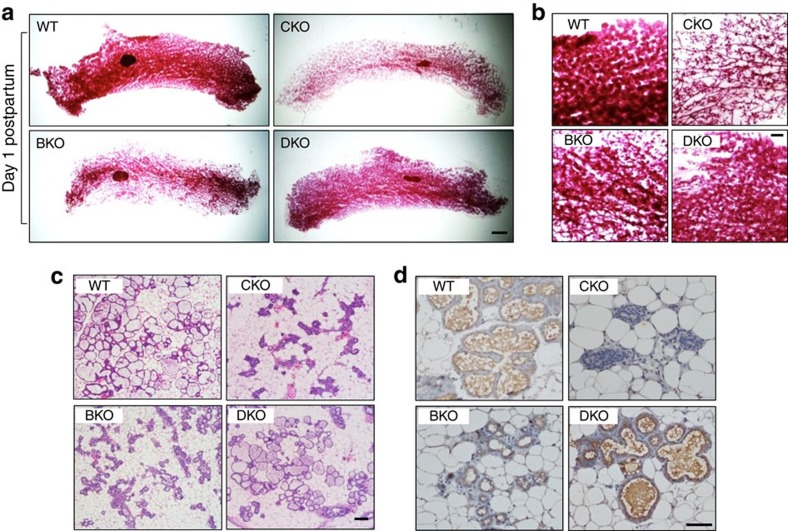
DKO rescues alveolar and lactogenic defects associated with CKO and BKO. (**a**,**b**) Whole mounts of mammary glands from 16 to 20-week mice 1-day postpartum. Scale bar, 1 mm in **a** and 500 μm in **b**. (**c**) H&E stain of the lobular-alveolar structure in mammary glands of 16–20-week mice 1-day postpartum. Scale bar, 100 μm. (**d**) Immunohistochemistry for total milk proteins in mammary glands of 16–20-week mice 1-day postpartum. Scale bar, 50 μm. Images in this figure are representatives of at least 4 animals in each genotype.

Despite the partial ductal growth in older virgin CKO (Fig. 1b), all pups of CKO dams died shortly after birth from obvious lack of nursing, suggesting a profound and persistent functional defect rather than transient ductal growth delay in CKO mammary glands. In support, mammary glands of CKO at postpartum were largely devoid of alveolar structure (Fig. 2a–c) and milk proteins (Fig. 2d). In contrast to CKO, hemizygous *Cobra1* knockout dams (*MMTV-Cre,Cobra1*^*f/+*^) displayed alveologenesis (Supplementary Fig. 2b) and nursing ability comparable to their WT littermates (*Cobra1*^*f/f*^). Thus, we conclude that COBRA1 is required for normal mammary gland functions.

Consistent with published reports^31^,^32^, we observed alveologenic and lactogenic defects in BKO mammary glands, albeit much milder than CKO (Fig. 2). In stark contrast, DKO dams with simultaneous deletion of *Brca1* and *Cobra1* underwent efficient alveologenesis and lactogenesis, as evidenced by the normal alveolar structure (Fig. 2a–c) and abundant milk proteins (Fig. 2d). Collectively, these genetic data unequivocally demonstrate a functional interaction between *Brca1* and *Cobra1* in mammary gland development and function.

### Gene-specific genetic interaction between *Brca1* and *Cobra1*

To determine how specific the genetic complementation is between *Brca1* and *Cobra1*, we first asked whether genetic ablation of other growth-arresting tumour suppressor genes could rescue the developmental defects associated with CKO. Tumour suppressor genes *Ink4-Arf* play a critical role in oncogene-induced senescence, and co-deletion of the *Ink4a/Arf* locus restored developmental defect associated with the loss of *Bmi1*, which encodes a transcriptional regulator of stem cell renewal^33^. Likewise, deletion of tumour suppressor gene *Trp53* partially rescued early embryonic lethality associated with *Brca1* deficiency^34^,^35^,^36^. We therefore combined CKO with whole-body deletion of *Ink4-Arf* or mammary gland-specific deletion of *Trp53*. In contrast to *Brca1* deletion, neither *Ink4-Arf* nor *Trp53* deficiency rescued the ductal growth defect of virgin CKO (Supplementary Figs 5 and 6), suggesting a gene-specific genetic interaction between *Brca1* and *Cobra1*. In a separate experiment, we did not find any sign of mutual rescue of embryonic lethality associated with *Brca1* or *Cobra1* deletion (Supplementary Table 1). Taken together, these findings underscore the specificity of the genetic interaction between *Cobra1* and *Brca1* in mammary epithelium.

### DKO exhibits altered epithelial homoeostasis

**Figure 3:**
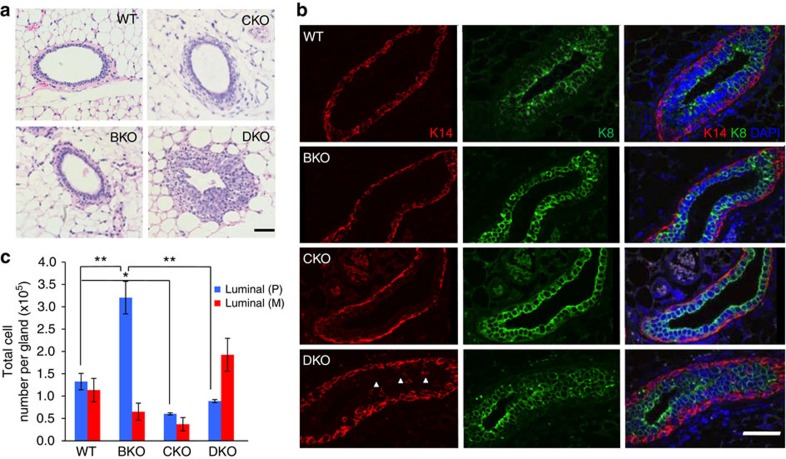
Altered epithelial homoeostasis in the absence of *Cobra1 and Brca1*. (**a**) H&E staining of mammary ducts from 8-week WT and KO animals. Scale bar, 50 μm. (**b**) Immunofluorescence staining with luminal epithelial and myoepithelial markers K8 and K14, respectively, from 8-week animals. Scale bar, 50 μm. (**c**) Enumeration of mature luminal (CD49f^med^EpCAM^high^CD49b^−^) and progenitor cells (CD49f^med^EpCAM^high^CD49b^+^). The numbers of animals used are: WT (4), BKO (3), CKO (3) and DKO (4). *P<0.05, **P<0.01 by Student's *t*-test. Error bars represent s.e.m.

While DKO had functionally restored mammary glands, its epithelial layers were appreciably thickened when compared with both WT and the single-gene KOs (Fig. 3a). This was largely due to an increased population of epithelial cells that expressed Keratin 8 (K8), an established luminal marker (Fig. 3b). In contrast, DKO still maintained a single layer of Keratin 14 (K14)-positive myoepithelial cells (Fig. 3b). However, we reproducibly observed a small number of K14-positive cells uniquely residing in the luminal epithelial compartment of DKO mammary ducts (arrows in Fig. 3b). Bromodeoxyuridine (BrdU) pulse-labelling and TUNEL analysis indicate both elevated cell proliferation and apoptosis rates, respectively, in DKO mammary epithelium (Supplementary Fig. 7), indicating an increased epithelial turnover. Thus, despite developmental and functional rescue, DKO mammary glands exhibit distinct epithelial cell dynamics and organization.

To assess further tissue homoeostasis in DKO mammary glands, we conducted flow cytometry to distinguish luminal progenitor cells from mature luminal cells in 8-week-old virgin animals after puberty, using CD49b as an established luminal progenitor marker^30^. Consistent with published findings^37^,^38^,^39^, we found that BKO had more luminal progenitor cells versus WT animals (Fig. 3c). In contrast, CKO mammary glands contained reduced pools of both mature (CD49b^−^) and progenitor (CD49b^+^) cells in the luminal epithelial compartment (Fig. 3c), again suggesting inhibition of mammary epithelial cells of all lineages and differentiation stages upon *Cobra1* ablation. Intriguingly, the flow cytometry profiles of DKO were distinct from those of BKO and CKO. In particular, the luminal progenitor cell population in DKO was substantially smaller than that in BKO (Fig. 3c). There was a concomitant upward trend in mature luminal cell abundance in DKO versus BKO (Fig. 3c). This result suggests that antagonism between BRCA1 and COBRA1 influences the relative abundance of mature and progenitor cells in the luminal compartment.

### DKO remains defective in HR-mediated DSB repair

**Figure 4:**
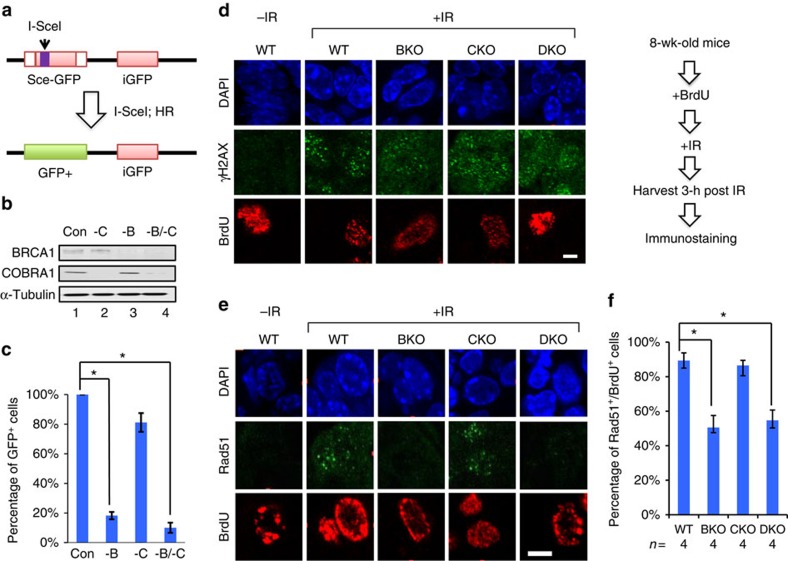
COBRA1 is not involved in BRCA1-mediated HR. (**a**) Diagram of the GFP reporter assay for measuring HR efficiency. I-Scel: restriction enzyme. iGFP: internal GFP fragment as the template for HR. (**b**) Immunoblot of COBRA1 and BRCA1 for assessing siRNA knockdown efficiency with control (Con) oligos or ones targeting human BRCA1 (-B) and COBRA1 (-C) in HeLa cells. (**c**) Percentage of GFP^+^ cells as a result of HR-mediated DSB repair. Results are average of three independent experiments, and were normalized to the control group. *P<0.05 by Student's *t*-test. (**d**) Mice of 8-wk old were pulse-labelled with BrdU, irradiated (20 Gy), and mammary glands were harvested 3 h later for immunostaining for γH2AX and BrdU. Scale bar, 5 μm. (**e**) The same samples as shown in **d** were stained for Rad51 and BrdU. Scale bar, 5 μm. (**f**) Percentage of Rad51^+^/BrdU^+^ mammary epithelial cells. *P<0.05 by Student's *t*-test. The numbers of animals used are indicated below the graph. Error bars represent s.e.m. wk, week.

Given the established role of BRCA1 in HR-mediated DSB repair, we asked whether the *Brca1*/*Cobra1* interaction affected DSB repair efficiency. First, we used a green fluorescence protein (GFP)-based reporter assay *in vitro*, in which repair of site-specific DSB through the HR-dependent pathway gives rise to a functional GFP gene^40^ (Fig. 4a). As expected, short interfering RNA (siRNA)-mediated knockdown of BRCA1 in HeLa cells significantly compromised HR efficiency, as indicated by the reduced percentage of GFP^+^ cells (Fig. 4b,c; Supplementary Fig. 8). Depletion of COBRA1 alone did not affect HR efficiency, nor did it rescue the HR defect in BRCA1-depleted cells (Fig. 4b,c; Supplementary Fig. 8), suggesting that COBRA1 was not involved in BRCA1-mediated DSB repair *in vitro*.

Next, we examined HR efficiency *in vivo* following ionizing radiation. HR repair predominantly occurs in proliferating cells during late S and G2 phases of the cell cycle, when sister chromatids are available as the homologous templates for HR-mediated repair^41^. We therefore tracked proliferating cells in irradiated mice by pulse labelling them with BrdU. DSB damage was monitored 3 h after irradiation by immunofluorescence staining for γH2AX. As expected, ionizing radiation-induced γH2AX nuclear foci were present in both BrdU^+^ and BrdU^−^ cells of WT and KO animals (Fig. 4d). To assess efficiency of HR-dependent DSB repair, we enumerated BrdU^+^ mammary epithelial cells with ionizing radiation-induced nuclear foci of the well-established HR marker Rad51. In particular, published studies demonstrated that recruitment of Rad51 to DSB sites is facilitated by BRCA1 (refs 42, 43). Consistent with the *in vitro* findings, irradiated BKO animals exhibited substantially lower Rad51^+^/BrdU^+^ ratios versus WT (Fig. 4e,f). CKO mammary glands had similar Rad51^+^/BrdU^+^ ratios versus WT control, again suggesting that COBRA1 is not required for ionizing radiation-induced DSB repair *per se*. Notably, HR repair in DKO mice remained as deficient as that in BKO (Fig. 4e,f). Taken together, the genetic complementation between *Brca1* and *Cobra1* does not affect DSB repair function of BRCA1.

### DKO has restored pubertal transcription

**Figure 5:**
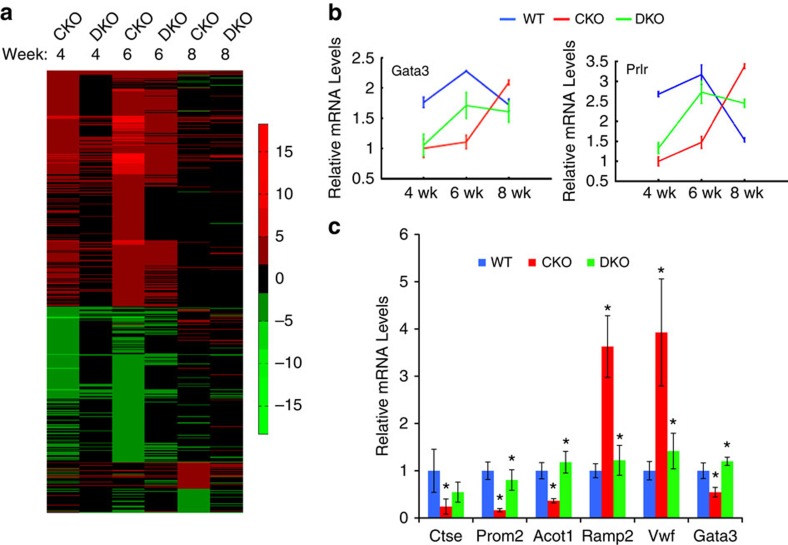
Aberrant pubertal gene expression in CKO is partially rescued in DKO. (**a**) Heatmap illustrates the gene expression changes in total mammary epithelial cells of CKO and DKO as compared with their corresponding WT littermates (*n*=3) at three time points (4, 6 and 8 wks). The gene expression levels in WT are set as 1. (**b**) Expression patterns for two representative pubertal genes that are affected by CKO and partially rescued by DKO. The lowest expression level in each graph is set at 1. (**c**) Confirmation of the microarray data by gene-specific RT-PCR for a number of pubertal genes, using RNA samples from 6-wk old mice. The result is average of values from 3 animals in each genotype. β-Actin mRNA was used for normalization. *P<0.05 by Student's *t*-test. Statistical analysis was conducted between WT and CKO, and between CKO and DKO. Error bars represent s.e.m. wk, week.

To gain molecular insight into the *Brca1*/*Cobra1* genetic complementation during ductal development at puberty, we carried out gene expression profiling of total mammary epithelial cells from virgin WT, BKO, CKO and DKO at 4, 6 and 8 weeks. Consistent with their normal ductal growth (Fig. 1a,b) and previously reported gene expression profiling of the same animal model^32^, BKO mice exhibited relatively few transcriptionally affected genes compared with their WT controls (Supplementary Data 1). In contrast, the gene expression profiles of CKO were significantly different from their WT littermates, with the most significant transcriptional aberration observed at the early (4 week) and mid-pubertal (6 week) stages (Fig. 5a; Supplementary Data 1). Furthermore, these CKO-affected genes were enriched with previously identified pubertal genes^44^ (*P*=7.65 × 10^−13^ for 6 weeks by Fisher's exact test), and oestrogen (*P*=7.73 × 10^−6^ by Fisher's exact test) and progesterone-responsive genes^45^ (*P*=5.00 × 10^−5^ by Fisher's exact test) in mammary epithelium (Supplementary Data 2). Strikingly, ∼80% of the CKO-affected genes at 4 and 6 weeks were either partially or completely rescued in DKO mammary glands (Fig. 5a; Supplementary Data 2). Likewise, the DKO-rescued genes were enriched with puberty-related (*P*=2.34 × 10^−9^ for 6 weeks by Fisher's exact test) and oestrogen (*P*=2.09 × 10^−5^ by Fisher's exact test) and progesterone-responsive genes (*P*=7.64 × 10^−4^ by Fisher's exact test, Supplementary Data 2). For example, expression of *Gata3* and *Prlr*, two known pubertal genes, was disrupted by *Cobra1* ablation but partially restored in DKO (Fig. 5b). We also confirmed the microarray result for several pubertal genes by gene-specific RT-PCR (PCR with reverse transcription; Fig. 5c). Of note, while the transcriptional rescue in DKO occurred as early as 4 weeks (Fig. 5a), restoration of ductal growth in DKO was not apparent until 8 weeks (Fig. 1a,b; Supplementary Fig. 4), likely due to incomplete transcriptional rescue of CKO-affected genes. The fact that transcriptional rescue precedes developmental rescue suggests that the former is likely a cause, rather than consequence, of the restored ductal morphogenesis. Collectively, the antagonistic activity of BRCA1 in pubertal gene expression provides a reasonable explanation for its development-arresting function in CKO mammary glands.

## Discussion

Our mouse genetic study unequivocally demonstrates an important role of the COBRA1 in mammary gland development. Furthermore, our work uncovers genetic complementation between *Brca1* and *Cobra1* in the context of mammary gland development. Historically, genetic suppression analysis has provided a highly valuable tool used in various simple model organisms for identifying functional interactions between genes that act in a common pathway or two related ones^46^. Although multiple mechanisms can explain a genetic suppression phenotype, the specific genetic complementation between *Brca1* and *Cobra1* is most likely due to an antagonistic action of these two gene products in a common pathway. First of all, the observed genetic suppression during DKO mammary gland development is associated with a similar functional rescue in pubertal transcriptional programme, but not in DSB repair. Second, mutual suppression, which was observed in alveologenesis and lactogenesis of DKO mammary glands (Fig. 2), often occurs between two genes that encode interacting proteins^46^. In this regard, the genetic finding serves as a satisfying functional validation of our previously characterized physical interaction between human BRCA1 and COBRA1 (ref. 11). Lastly, CKO-associated developmental defects were only suppressed by deletion of *Brca1*, not other tumour suppressor genes tested, including *Trp53*. This gene-specific genetic suppression most likely reflects a COBRA1-opposing action of BRCA1 in a specific functional pathway, rather than a more general, p53-like DNA damage checkpoint function in cell cycle arrest and/or apoptosis.

The *MMTV-Cre* system has been widely used in tissue-specific characterization of genes involved in mouse mammary gland development and functions^47^,^48^. However, recent reports indicated that *MMTV-Cre* Line A, which was used in the current study, exhibited moderate impairment in alveologenesis and lactogenesis^49^,^50^. This reported side effect of *MMTV-Cre* unlikely affects our conclusions on BRCA1 and COBRA1 for the following reasons. First, unlike age-matched CKO, hemizygous *MMTV-Cre,Cobra1*^*f/+*^ dams displayed grossly normal alveologenesis and nursing capability. In contrast to the previous reports^49^,^50^, the absence of any appreciable *MMTV-Cre*-associated lactational defects in our study could be due to the more heterogeneous strain background in our work. Furthermore, virgin CKO animals, but not *MMTV-Cre,Cobra1*^*f/+*^, display a dramatic defect in mammary ductal development at all age groups examined (6–24 weeks of age, Fig. 1a,b). In this regard, no ductal defects were reported for virgin *MMTV-Cre* Line A animals in previously published studies^49^,^50^. Lastly, CKO, BKO and DKO, which were assessed in the same *MMTV-Cre* Line A background, gave rise to distinct alveologenic and lactogenic phenotypes. Taken together, these data strongly suggest that usage of *MMTV-Cre* Line A alone is not the cause of the profound developmental defects in CKO or the striking genetic rescue observed in DKO.

Our genetic findings clearly indicate that DNA repair-independent antagonism between BRCA1 and COBRA1 plays a critical role in pubertal mammary gland development and maintenance of mammary epithelial homoeostasis. Loss of COBRA1 manifests the BRCA1-mediated inhibition of ductal morphogenesis as observed in CKO, and conversely, BKO mice without functional BRCA1 exhibit enlarged luminal progenitor cell population and defective alveolar development as a result of unopposed COBRA1 actions. Absence of both BRCA1 and COBRA1 in DKO mammary glands could reach a quasi-balanced state that allows for gross tissue development and restored mammary gland functions. While functions of DKO mammary glands are largely restored to a level comparable to WT, the inner layer of DKO epithelial ducts tend to have an expanded K8^+^ cell population juxtaposed with some K14^+^ cells. Furthermore, the ratio of CD49b^+^ over CD49b^−^ luminal cells in DKO is significantly lower in DKO versus WT. Lastly, DKO epithelium experiences both increased proliferation and apoptosis, which could explain why the absolute number of total live cells as measured by flow cytometry is similar to that in WT animals (Fig. 1c). We speculate that DKO luminal epithelium may have the propensity for precocious differentiation, thus resulting in exhaustion of the luminal progenitor subpopulation. As luminal progenitor cells are thought to be the cells of origin for BRCA1-associated tumours^37^,^38^,^39^, investigation is under way to interrogate the impact of *Cobra1* ablation on tumorigenesis in *Brca1*-deficient mouse mammary epithelium. Our current genetic work underscores the importance of interrogating functional interactions between *Brca1* and *Cobra1* in the physiologically relevant tissue context and developmental window.

## Methods

### Mice

*Cobra1*^*f/f*^ mice have been described previously^26^. *MMTV-Cre,Cobra1*^*f/f*^ mice were generated by breeding *MMTV-Cre* Line A animals (from Dr Anthony Wynshaw-Boris) with *Cobra1*^*f/f*^ mice. *Trp53*^*f/f*^ (Trp53tm1Brn), *Ink4-Arf KO*, and *Brca1*^*f/f*^ mice^31^ were obtained from Mouse Model of Human Cancer Consortium (MMHCC), National Cancer Institute. EIIa-Cre was purchased from the Jackson Laboratory, and used to generate the whole-body hemizygous deletion strain *Brca1*^*+/−*^*,Cobra1*^*+/−*^ per previously described procedures^26^. All mouse strains were in a similarly mixed genetic background (129SvEv/SvJae/C57BL6/FVB). Mutant mice were analysed with their corresponding littermate controls. All procedures performed on animals were in compliance with ethical regulations and were approved by the Institutional Animal Care and Use Committee (IACUC) at the University of Texas Health Science Center at San Antonio.

### Whole mount and immunostaining

Inguinal mammary glands from mice of different age groups as indicated in individual figures were used for whole-mount staining. Inguinal fat pads were gently isolated and spread onto a glass slide. Glands were fixed in Carnoy's fixative (ethanol:chloroform:glacial acetic acid, 60:30:10) overnight at room temperature. Glands were rehydrated in descending grades of alcohol (70, 50 and 30%) for 15 min each, then washed with distilled water before overnight staining in Carmine alum (1 g carmine, 2.5 g aluminium potassium sulfate boiled for 20 min in distilled water, filtered and brought to a final volume of 500 ml). Stained glands were dehydrated in ascending grades of alcohol (70, 70, 90, 95, 100 and 100%) for 15 min each, and cleared with Citrisolv reagent (Fisher, Cat no. 22-143975). Samples were examined under a Nikon SMZ1000 dissection microscope. Duct length was measured from calibrated images using Eclipse software. Average length of three longest ducts from nipple region in inguinal mammary glands was taken as the ductal length of each animal. The value shown in Fig. 1b is mean of ductal lengths for multiple animals of a given genotype at a given age±s.e.m.

Primary antibodies used were anti-COBRA1 (1:50) (ref. 26), anti-milk protein (Nordic Immunology, RAM/MSP, 1:10,000), anti-K8 (Developmental Studies Hybridoma Bank, TROMA-1, 1:100), anti-K14 (Covance, PRB-155P, 1:5,000), anti-BrdU (GE Healthcare, RPN20, 1:10,000), anti-γH2AX (Cell Signaling, 9718, 1:500), and anti-Rad51 (Santa Cruz, sc-8349, 1:100).

Mammary glands were fixed in 10% neutral buffered formalin for 18 h at 4 °C and paraffin embedded. Sections of 2 or 3 μM in thickness were used for hematoxylin–eosin (H&E) staining, immunofluorescent staining and immunohistochemistry. Samples were baked at 70 °C for 15 min, then de-paraffinized by three 5-min extractions in 100% xylene, followed by 3-min each of descending grade of alcohol (100% twice, 95, 70 and 50%). Samples were washed briefly with phosphate-buffered saline (PBS) before transferring to boiling antigen-unmasking solution (Vector Labs, H-3300) for 20 min. For immunohistochemistry, sections were pre-treated with 3% hydrogen peroxide for 10 min before blocking. Blocking was performed with 10% normal goat serum in PBS for 1 h at room temperature followed by primary antibody incubation overnight at 4 °C. For detection with primary antibody using the immune enzymatic method, the ABC peroxidase detection system (Vector Labs, PK-6105) was used with 3, 3′-diaminobenzidine (DAB) as substrate (Vector Labs, SK-4105) according to manufacturer's instruction.

For immunofluorescence staining, sections were incubated with Alexa-488 and Alexa-546-conjugated secondary antibodies (Life Technologies), mounted with Vectashield mounting medium with DAPI (Vector Labs, H-1200), and examined with an Olympus FV1000 confocal microscope or Nikon Eclipse Ni fluorescent microscope. For BrdU pulse-labelling, mice were intraperitoneally injected with cell proliferation labelling reagent (GE Healthcare, RPN201) at 16.7 ml kg^−1^. For BrdU/Rad51 and BrdU/γΗ2ΑX double staining, mice were first injected with BrdU and then X-rayed at 20 Gy using a Faxitron cabinet X-ray system (Model 43855F). Mammary glands were harvested 3 h after labelling.

### Flow cytometry

Thoracic and inguinal mammary glands from virgin mice were isolated in sterile condition and lymph nodes from inguinal glands were removed. Single cells were prepared using published protocol^51^ with minor modifications. All reagents were purchased from StemCell Technologies (Vancouver, Canada), unless otherwise indicated. Briefly, isolated glands were minced using scissors and digested for 15–18 h at 37 °C in DMEM F-12 (Cat. no. 36254) containing 2% fetal bovine serum (FBS), insulin (5 mg ml^−1^), penicillin–streptomycin and a final concentration of 1 mg ml^−1^ collagenase and 100 U ml^−1^ hyaluronidase (Cat. no. 07919). After vortexing, epithelial organoids were collected by centrifugation at 600*g* for 4 min. Red blood cells (RBCs) in the resulting pellets were lysed with 0.8% NH_4_Cl. Epithelial organoids were then digested by pipetting with 2 ml of 0.05% pre-warmed Trypsin (Life Technologies, 25300) for 2 min, followed by washing in ice-cold Hanks Balanced Salt Solution (Cat. no. 37150) with 2% FBS (HF). Cells were resuspended in 5 mg ml^−1^ Dispase (Cat. no. 07913) with 0.1 mg ml^−1^ DNAse I (Sigma-Aldrich, D4513). After trituration for 1–2 min, cells were resuspended in ice-cold HF, and single cells were prepared by filtering the cell suspension through a 40-μm cell strainer (Fisher, Cat. no. 22363547). Cells were counted and resuspended in HF at a concentration of 1 × 10^6^ cells per 100 μl. Cell were incubated for 10 min on ice with 10% rat serum (Jackson Laboratories, 012-000-120) and Fc receptor antibody (BD Biosciences, 553141). After blocking, cells were incubated for 20 min with antibodies for the following cell-surface markers: Ep-CAM-PE (BioLegend, 118206, 0.5 μl per 100 μl), CD49f-FITC (BD Biosciences, 555735, 2 μl per 100 μl), CD31-Biotin (BD Bioscience, 553371, 1 μl per 100 μl), CD45 biotin (BioLegend, 103103, 1 μl per 100 μl), TER-119 Biotin (BioLegend, 103511, 1 μl per 100 μl), and CD49b-Alexa Fluor 647 (BioLegend, 104317, 0.5 μl per 100 μl) followed by Streptavidin-Pacific Blue (Invitrogen, S11222, 0.5 μl per 100 μl) incubation. 7-AAD (BD Biosciences, 559925, 5 μl per 100 μl) was added 10 min before analysis. CD49b^+^ cells were gated using a fluorescent-minus-one control, in which all antibodies except CD49b-Alexa 647 were used. Sorting was performed with a Moflo Astrios cell sorter (Beckmen Coulter). Data were analysed using FACSDiva software. Purity of the stromal, luminal, and myoepithelial populations were verified by RT-PCR analysis of Vimentin (stromal), K18 (luminal), K5 (myoepithelial) and K14 (myoepithelial) mRNA.

### *In vitro* HR-based DSB repair assay

The homology-directed repair assay was performed using established methods^52^. The recombination substrate, pDR-GFP, contains two inactive GFP genes, one of which is due to the presence of an I-SceI endonuclease recognition sequence. This DNA is integrated into a single site in HeLa cells. On day 1, siRNAs specific for a control sequence, COBRA1, and BRCA1 were transfected, using Oligofectamine (Invitrogen), into wells containing HeLa-DR-GFP cells. On day 3, the cells were re-transfected with the same siRNAs plus a plasmid for the expression of the I-SceI endonuclease using the Lipofectamine 2000 transfection reagent (Invitrogen). On day 6, cells were released from the monolayer using trypsin and the fraction of GFP^+^ cells was determined using a FACSCalibur analytical flow cytometry instrument. Results were normalized to the per cent of GFP^+^ cells in the sample in which the control siRNA was transfected.

### Gene expression profiling and bioinformatics analysis

Triplicates of RNA samples from live Lin^-^/CD24^+^ mammary epithelial cells of WT and mutant mice were labelled using the Illumina TotalPrepTM RNA amplification kit (Ambion, Cat. no. AMIL1791) and subsequently hybridized to Illumina mouse whole genome gene expression BeadChips (MouseRef-8 version 2.0, Illumina). BeadChips were scanned on an iScan Reader (Illumina) using iScan software (version 3.3.29, Illumina). For further analysis, the scanned data were uploaded into GenomeStudio software (version 1.9.0, Illumina) via the gene expression module (Direct Hyb). The genomic data are available at NIH Gene Expression Omnibus site (GSE67440).

For each of the time points, we identified genes that are affected by *Cobra1* KO (CKO-affected) and those that are eventually rescued by double KO (DKO-rescued). We define CKO-affected genes as the genes that show ≥2.0 fold enrichment (either up or down) in CKO mice compared with corresponding WT control mice, with *P*≤0.05. DKO-rescued genes are defined as those CKO-affected genes that had either (1) ≤1.5 fold enrichment (either up or down, *P*<0.05) in DKO versus WT control mice, or (2) fold of changes in DKO versus WT (*P*<0.05) no more than 50% of those in CKO versus WT, or (3) any fold of changes in DKO versus WT with *P* value larger than 0.05. Supplementary Data 2 shows the total number of CKO-affected and DKO-rescued genes for the indicated time points.

Pubertal, oestrogen and progesterone signature genes were extracted from previously published studies^44^,^45^ to identify the overlap with CKO-affected or DKO-rescued genes. Supplementary Data 2 shows the overlap among CKO-affected/DKO-rescued genes with pubertal, oestrogen and progesterone genes. The statistical significance (*P* value) of the overlap was calculated using the Fisher's exact test:


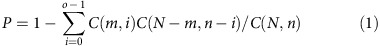


where *N* is the total number of genes in the experiment; *m,n* is the selected affected/rescued and previously published signature genes respectively and *o* is the overlap among those genes. *C*(*n,k*) is the binomial coefficient.

The microarray data have been submitted to the NIH database (accession number GSE67440).

### Primer sequences

For RT-PCR: 18sRNA-F: 5′-GAATTCCCAGTAAGTGCGGG-3′, 18sRNA-R: 5′-GGGCAGGGACTTAATCAACG-3′. Cobra1-F: 5′-ACAACTTCTTCAGCCCTTCCC-3′, Cobra1-R: 5′-TCTGCACCACCTCTCCTTGG-3′. Brca1-F: 5′-AGCAAACAGCCTGGCATAGC-3′, Brca1-R: 5′-ACTTGCAGCCCATCTGCTCT-3′. p16Ink4a-F: 5′-GAACTCTTTCGGTCGTACCCC-3′, p16Ink4a-R: 5′-CGTGAACGTTGCCCATCAT-3′. p19Arf-F: 5′-CTTGAGAAGAGGGCCGCAC-3′, p19Arf-R: 5′-AACGTTGCCCATCATCATCA-3′. p53-F: 5′-GAGACAGCAGGGCTCACTCC-3′, p53-R: 5′-TGGCCCTTCTTGGTCTTCAG-3′. Ctse-F: 5′-ATTGGCAGATTGCCCTGGAT-3′, Ctse-R: 5′-GCCTTCGGAGCAGAACATCA-3′. Prom2-F: 5′-TGACCTGGATAAGCACCTGG-3′, Prom2-R: 5′-AAGCTCTGAAGCTCCTGCTG-3′. Acot1-F: 5′-ATGGCAGCAGCTCCAGACTT-3′, Acot1-R: 5′-CCCAACCTCCAAACCATCAT-3′. Ramp2-F: 5′-GCCTCATCCCGTTCCTTGTT-3′, Ramp2-R: 5′-CCTGGGCATCGCTGTCTTTA-3′. Vwf-F: 5′-CGACCTGGAGTGTATGAGCC-3′, Vwf-R: 5′-ACACACTTGTTTTCGTGCCG-3′. Gata3-F: 5′-GATGTAAGTCGAGGCCCAAG-3′, Gata3-R: 5′-GCAGGCATTGCAAAGGTAGT-3′. K18-F: 5′- ACTCCGCAAGGTGGTAGATGA-3′, K18-R: 5′- TCCACTTCCACAGTCAATCCA-3′, K14-F: 5′- AGCGGCAAGAGTGAGATTTCT-3′, K14-R: 5′- CCTCCAGGTTATTCTCCAGGG -3′, K5-F: 5′-GAGATCGCCACCTACAGGAA-3′, K5-R: 5′-TCCTCCGTAGCCAGAAGAGA-3′, Vimentin-F: 5′-CGGCTGCGAGAGAAATTGC-3′, Vimentin-R: 5′- CCACTTTCCGTTCAAGGTCAAG-3′, β-Actin-F: 5′-CGGTTCCGATGCCCTGAGGCTCTT-3′, β-Actin-R: 5′-CGTCACACTTCATGATGGAATTGA-3′.

### Statistical analysis

All data are expressed as arithmetic mean±s.e.m. Differences between relevant test and control group means were tested using an unpaired two-tailed Student's *t*-test unless otherwise indicated. For measures repeated at multiple ages, comparisons between test and control were performed on matched data from each time point. We considered a *P* value of<0.05 as statistically significant. Analytical approaches for microarray data are given in a separate section above.

## Additional information

**Accession codes:** The microarray data have been deposited in the NIH database under the accession number GSE67440.

**How to cite this article:** Nair, S. J. *et al*. Genetic suppression reveals DNA repair-independent antagonism between BRCA1 and COBRA1 in mammary gland development. *Nat. Commun.* 7:10913 doi: 10.1038/ncomms10913 (2016).

## Supplementary Material

Supplementary Information Supplementary Figures 1-8 and Supplementary Table 1

Supplementary Data 1 Lists of genes that are differentially expressed between wild-type control mice and mutant mice at 4, 6 and 8 weeks of ages.

Supplementary Data 2 Lists of total and puberty-related genes that are affected by CKO and rescued by DKO.
