# Supplementary material for: Genetic suppression reveals DNA repair-independent antagonism between BRCA1 and COBRA1 in mammary gland development
Source: Nat Commun. 2016 Mar 4;7:10913. doi: 10.1038/ncomms10913 (PMC4785232; doi:10.1038/ncomms10913)
Supplement: Supplementary Information — Supplementary Figures 1-8 and Supplementary Table 1 [file ncomms10913-s1.pdf]

Supplementary Figure 1. Deletion of *Brca1* and *Cobra1* in mouse mammary epithelium.

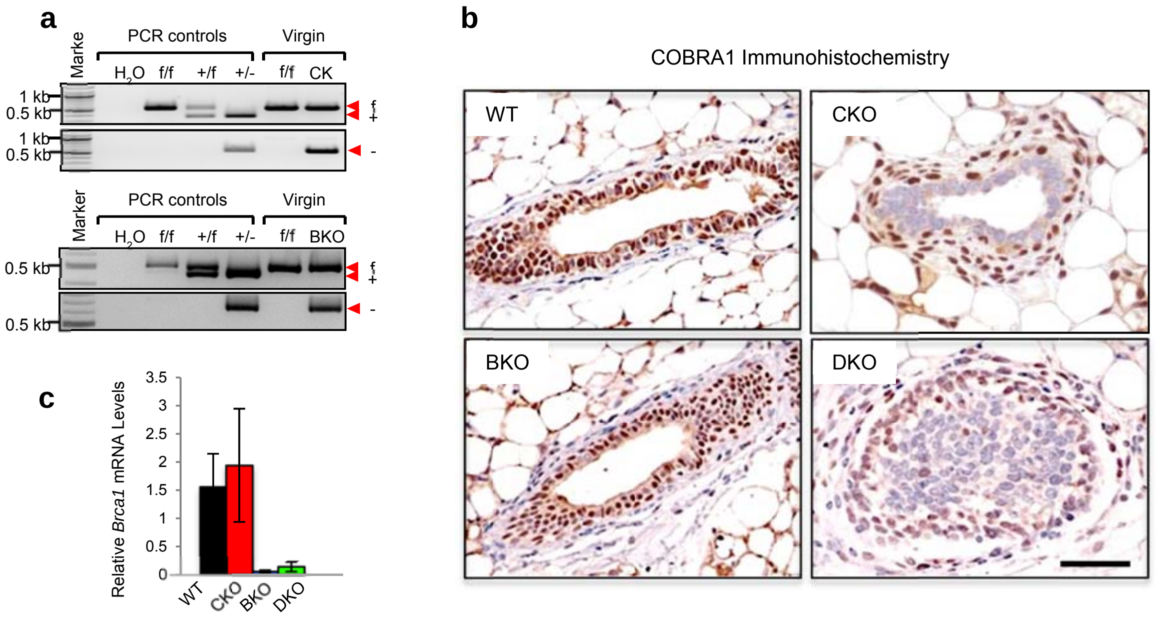

Supplementary Figure 1. Deletion of *Brca1* and *Cobra1* in mouse mammary epithelium. (***a***) Representative genotyping results for WT (+), floxed (f), and deleted (-) alleles of *Cobra1* (top) and *Brca1* (bottom) in mammary glands of control and mutant mice. Specific genotyping conditions will be provided upon request. (***b***) COBRA1 IHC in 8-week virgin mice. Representative results from at least 5 sets of animals. Scale bar: 50 µm. (***c***) RT-PCR analysis of *Brca1* mRNA levels from sorted luminal mammary epithelial cells (n=3). 18S rRNA was used for normalization. Error bars represent standard error of the mean (s.e.m.).

Supplementary Figure 2. Homozygous *Cobra1* deletion results in defects in ductal growth and alveologenesis.

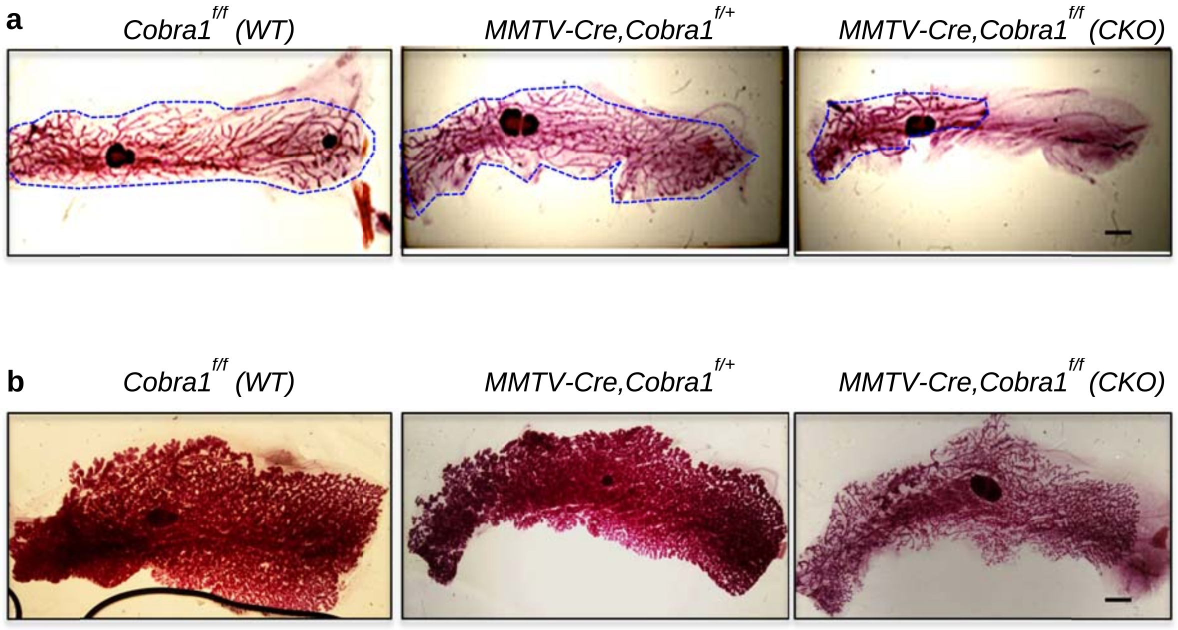

Supplementary Figure 2. Homozygous *Cobra1* deletion results in defects in ductal growth and alveologenesis. (***a***) Whole mounts of 8-wk virgin mice. (***b***) Whole mounts of 20-wk mice one day postpartum. Representative images from at least 4 animals in each genotype. Scale bar: 1 mm.

Supplementary Figure 3. Sorting of luminal and myoepithelial cells.

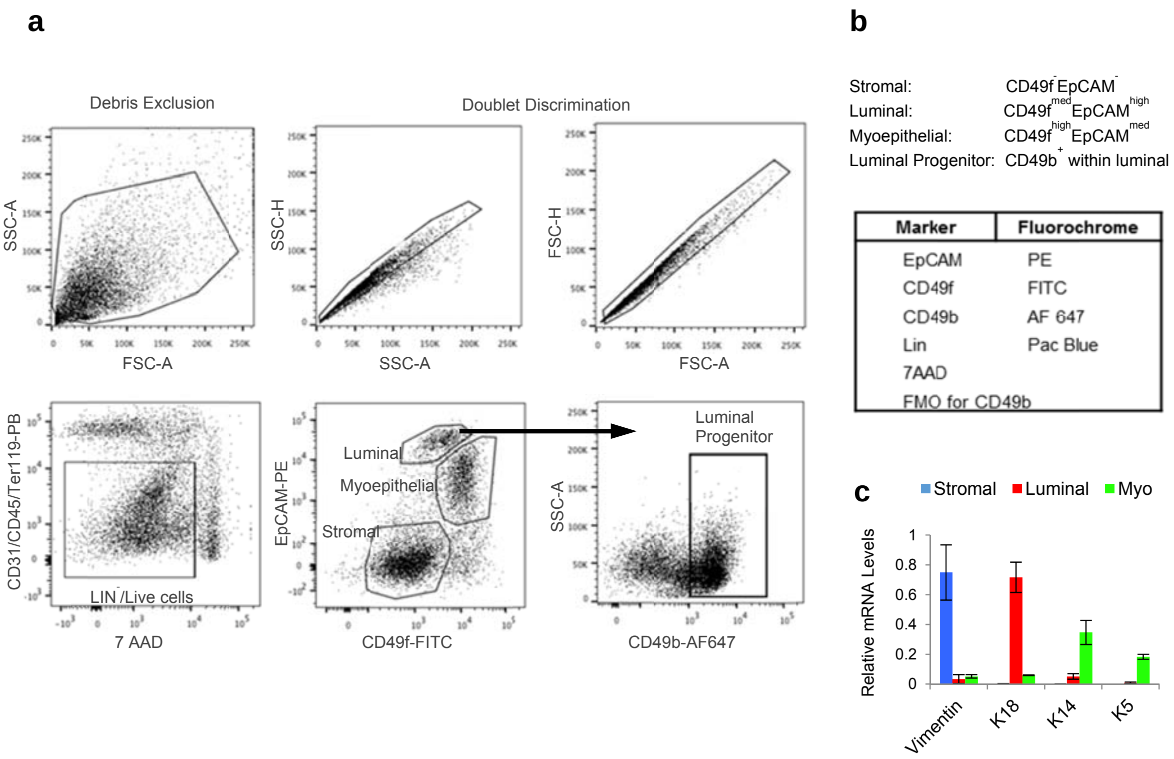

Supplementary Figure 3. Sorting of luminal and myoepithelial cells. (***a***) Representative flow cytometry results indicating the typical gating for debris exclusion, doublet discrimination, selection of lineage-negative/live cells, and separation of luminal, myoepithelial cells and stromal cells. (***b***) Cell surface markers and fluorochromes used in the flow cytometry. (***c***) Validation of cell sorting efficiency by RT-PCR of known stromal (Vimentin), luminal (K18), and myoepithelial cell (K5 and K14) markers (n=3). β-Actin mRNA was used as for normalization. Error bars represent s.e.m.

Supplementary Figure 4. Lack of signs of morphogenic rescue in 6-week old DKO.

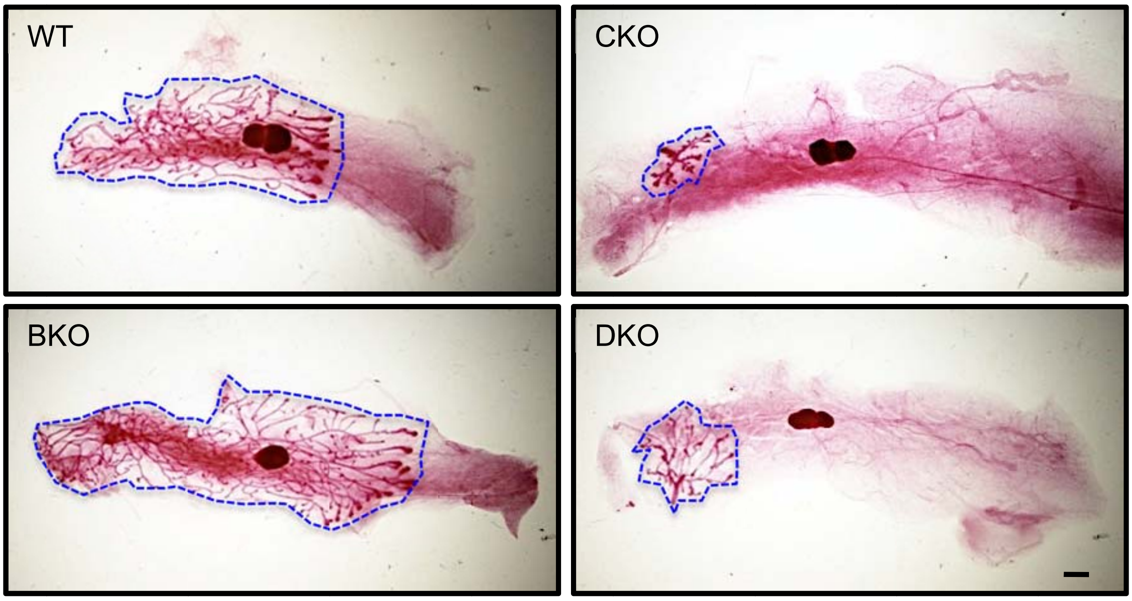

Supplementary Figure 4. Lack of signs of morphogenic rescue in 6-week DKO. Whole mounts of mammary gland tissue from 6-week animals. The images are representatives of at least 3 animals in each genotype. Scale bar: 1 mm.

Supplementary Figure 5. Developmental defect in CKO cannot be rescued by *Ink4-Arf* deletion.

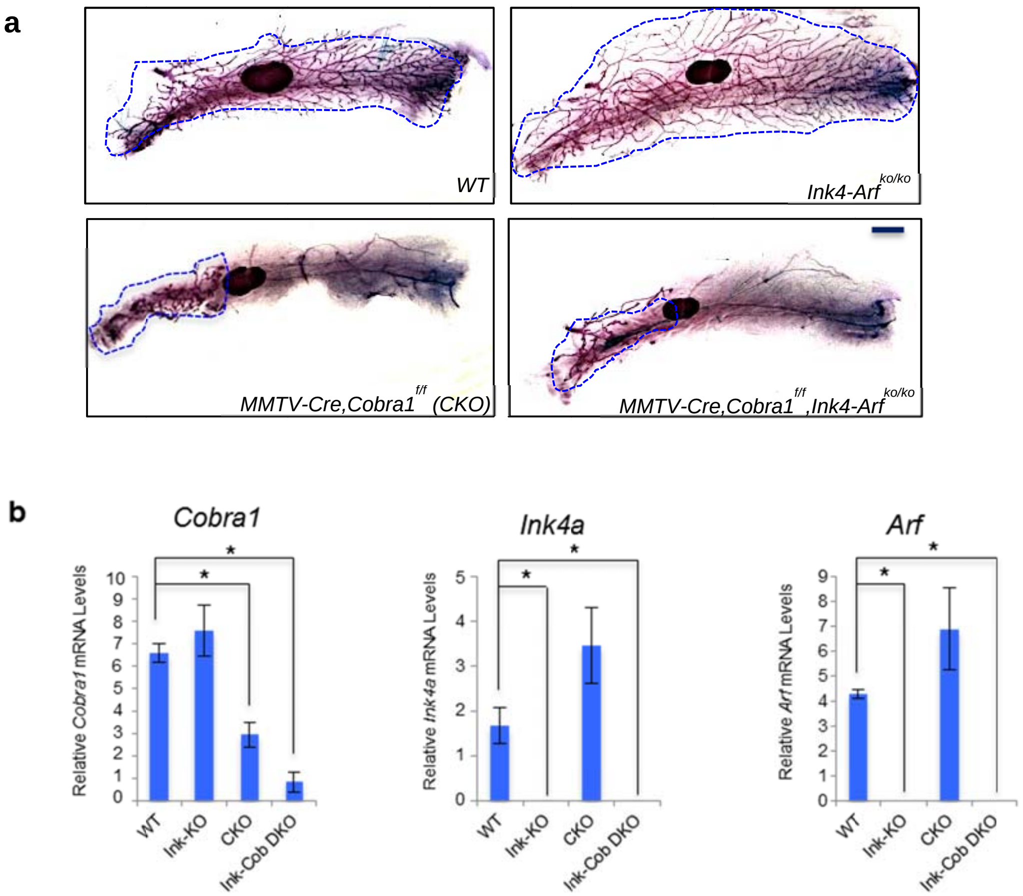

Supplementary Figure 5. Developmental defect in CKO cannot be rescued by *Ink4-Arf* deletion. (***a***) Whole mounts of 8-wk virgin mice. Representative images from at least 3 mice in each genotype group. Scale bar: 1 mm. (***b***) mRNA analysis for *Cobra1*, *Ink4a*, and *Arf* by RT-PCR, using sorted total mammary epithelial cells (n=3). 18S rRNA was used as the normalization control. * $P<0.05$ by Student's *t*-test. Error bars represent s.e.m.

Supplementary Figure 6. Developmental defect in CKO cannot be rescued by *Trp53* deletion.

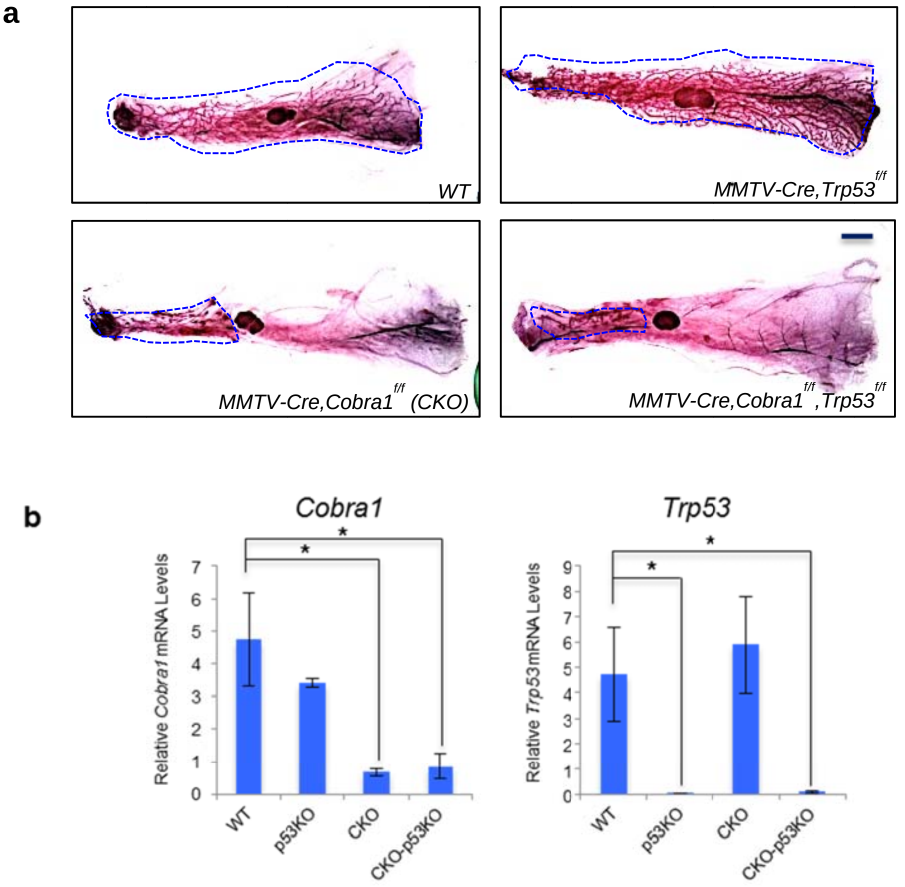

Supplementary Figure 6. Developmental defect in CKO cannot be rescued by *Trp53* deletion. (***a***) Whole mounts of 8-wk virgin mice. Representative images from at least 3 mice in each genotype group. Scale bar: 1 mm. (***b***) mRNA analysis for *Cobra1* and *Trp53*, using sorted total mammary epithelial cells (n=3). 18S rRNA was used as the normalization control. * P<0.05 by Student's t-test. Error bars represent s.e.m.

Supplementary Figure 7. Cell proliferation and apoptosis in WT and mutant mammary epithelium.

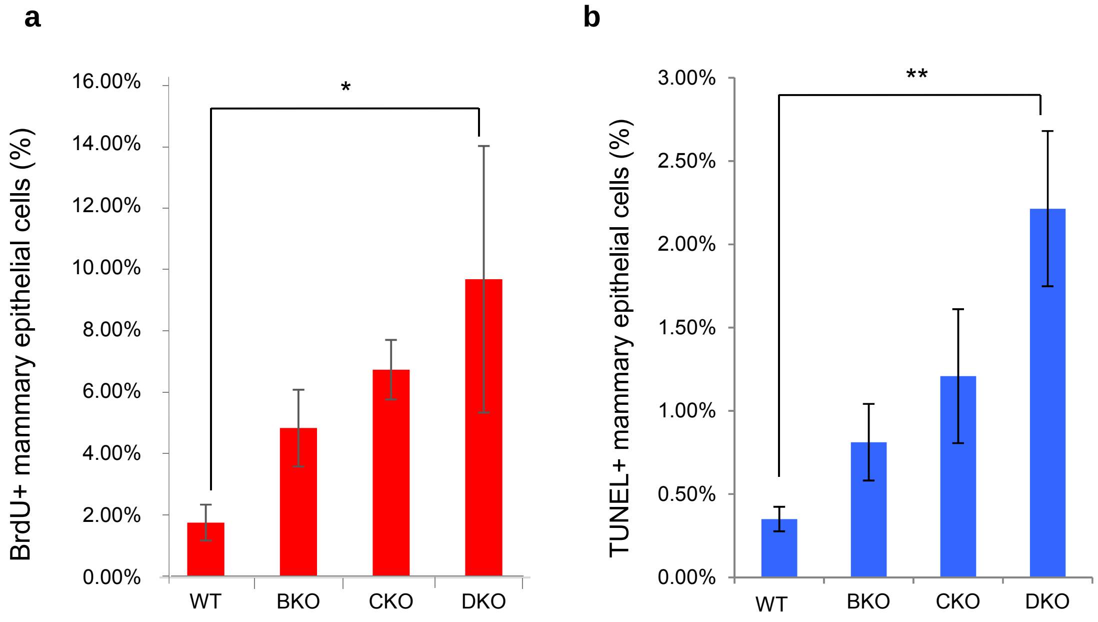

Supplementary Figure 7. Cell proliferation and apoptosis in WT and mutant mammary epithelia. (***a***) Percentage of BrdU+ mammary epithelial cells after 3-hour pulse labeling in 8-wk virgin WT (n=5), BKO (n=5), CKO (n=3), and DKO (n=3) mutant animals. (***b***) Percentage of TUNEL+ cells in mammary epithelium. Five animals were used for each of the four genotypes. * P<0.05, ** P<0.01 by Student's *t*-test. Error bars represent s.e.m.

Supplementary Figure 8. Full Western images related to Figure 4b.

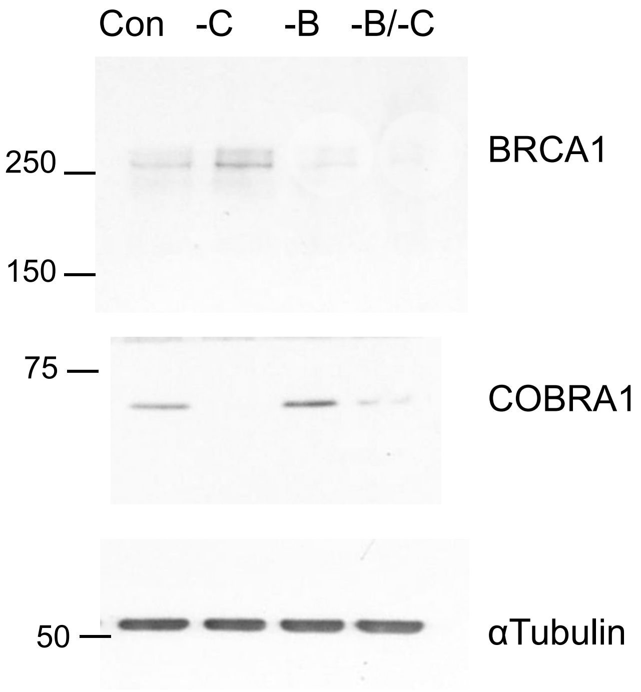

Supplementary Figure 8. Uncropped immunoblots for human BRCA1, COBRA1, and α-tubulin as shown in Figure 4b. Also shown on the left are molecular markers (kilodalton or kD)

Supplementary Table 1. Lethality of *Brca1*- and *Cobra1*-deleted embryos cannot be mutually rescued by double KO. Shown are numbers of viable progenies with various genotypes.

**Brca1,Cobra1**	**FEMALE**	**MALE**	**F + M**
**+/+, +/+**	11	20	31
**+/+, +/-**	25	18	43
**+/+, -/-**	0	0	**0**
**+/-, +/+**	35	23	58
**+/-, +/-**	69	55	124
**+/-, -/-**	0	0	**0**
**-/-, +/-**	0	0	**0**
**-/-, -/-**	0	0	**0**
**TOTAL**	140	116	256
